# Tracheotomy, and Permanent Breathing through a Tube

**Published:** 1831

**Authors:** 


### LXXIV.

TRACHEOTOMY, AND PERMANENT BREATHING THROUGH A TUBE. By Mr. J. KENWORTHY.

THE following very interesting case is extracted from a letter, lately received by the editor, from Mr. Kenworthy of Strangeways, Manchester.

*Case.* The subject was a *poor-house* patient in a district of the West Riding of Yorkshire. A young woman about 18 years of age. She had been under my care at intervals. Her complainings were symptoms of chronic inflammation of the glottis and trachea, which need not be defined—her own account was, that she swallowed a thimble some years before, and imagined the article still remained in the passage, as the cause of her distress. There was an enlargement externally, which gave her pain when examined, and a hard tumor presented itself to my finger down the anterior part of the œsophagus. Her breathing was difficult, and the difficulty had the appearance of being produced more by pressure than by the influence of inflammatory disturbance.

My opinion was that, eventually, laryngotomy would be necessary. Every means I could devise had been resorted to—bleeding, blistering, evacuations, antispasmodics, mercury to affect the glandular system had been used twice but without permanent benefit. I would not strictly say without benefit, for during the ptyalism, both times, the difficulty of respiration was lessened; but when the mercurial effect subsided, the same symptoms returned, and the latter time with greater violence. Her strength was now greatly reduced—her pulse feeble at 85, and her appetite diminished. On Sunday morning, May 24th, 1818, they came for me in haste, saying they believed her dying. I rode over as quickly as possible. Her situation was pitiable—the natural effort had arrived at a climax—she appeared fighting with death—her countenance livid and cadaverous—her pulse, from exhaustion and irregularity, not to be counted—there was no time to be lost—the first desideratum was relief to her breathing. I determined on Charles Bell's operation of laryngotomy, but think it extremely difficult, if not in this case impossible, as the whole of the larynx and trachea were drawn upwards and downwards with great force, in the exertions she made. I therefore extended the incision downwards, taking care to avoid the thyroid gland—opened the space between the sterno-hyoid and sterno-thyroid muscles, and dissected down to the trachea. I pushed a lancet into the front of the trachea, and, with a probe-pointed bistoury, divided downwards through three of the cartilaginous rings. The air instantly rushed out, throwing the blood in my face. My patient fainted, and remained in a faint state perhaps an hour. She was relieved from the urgency of her symptoms. When she was sufficiently recovered, by the use of stimulants, &c. I introduced my finger into the opening in front, upwards and also downward; but no extraneous substance was in the tube. The larynx was straitened so much as not to admit my little finger. I examined again by the mouth. The same tumour was there as before; so that I concluded the extraneous substance, if still remaining there, was lodged in the posterior part of the larynx, betwixt that and the pharynx The girl could not be left in her present state, as the object of the operation had not been fully obtained. I

therefore determined to dissect round to the back part of the trachea—it was a dreadful alternative, after the pain she had suffered; yet I thought it indispensable, and consequently made an incision by the anterior side of the sterno-cleido-mastoideus, parallel with the carotids to the posterior part of the larynx, and found, not a thimble or any other extraneous substance, but the cricoid cartilage enlarged and approaching to ossification—the case appeared hopeless.

June 21st. She remains in tolerable ease, but debilitated, and cannot bear to sit up. The discharge from the neck has been copious—occasioned perhaps by a silver wire doubled, which I introduced into the slit of the trachea, for want of a better instrument, and which I have since obtained from London, viz. a silver tube three-eighths of an inch diameter, with a shield in front and curved in the middle, for the purpose of extending a short way down the trachea.

Sept. 16th. Pulse 125; costive and hysterical—great pain in her head and sensibility of the eyes—thirsty and feverish. Gave her pil. fætid. and mist. aper. salin. 2a quaque hora.

18th. Better—pulse 115—had no evacuations from the bowels to day—spasms trouble her at times—continue ut antea.

19th. Much better—slept well—pulse 80, somewhat irregular—bowels open, and head easy—eyes not so sensible to the effects of light—had no spasm since last evening—there has been a considerable purulent discharge from the opening in front of the neck. Continue the medicine, pro re nata.

Dec. 27th. Another abscess has burst from the larynx, and she is now much better again.

1819, May 7th. She has generally been pretty well. I have occasionally visited her—the disturbed sensibility of the brain again shews itself—her breathing laboured and difficult—her pulse 110—feverish and restless—bowels confined. Mist. salina aperiens, and to breathe the vapour of warm water. A few days afterwards there was a purulent discharge again, but smaller in quantity—there was occasional necessity for caustic to fungous enlargements; but subsequently she arrived at a tolerable state of health and lived near eight years through the means of artificial respiration, through a tube.

